# Supplementary material for: Orientia tsutsugamushi Infection Stimulates Syk-Dependent Responses and Innate Cytosolic Defenses in Macrophages
Source: Pathogens. 2022 Dec 29;12(1):53. doi: 10.3390/pathogens12010053 (PMC9861896; doi:10.3390/pathogens12010053)
Supplement: Supplementary file 1 [file pathogens-12-00053-s001.zip › pathogens-2068194-supplementary.pdf]

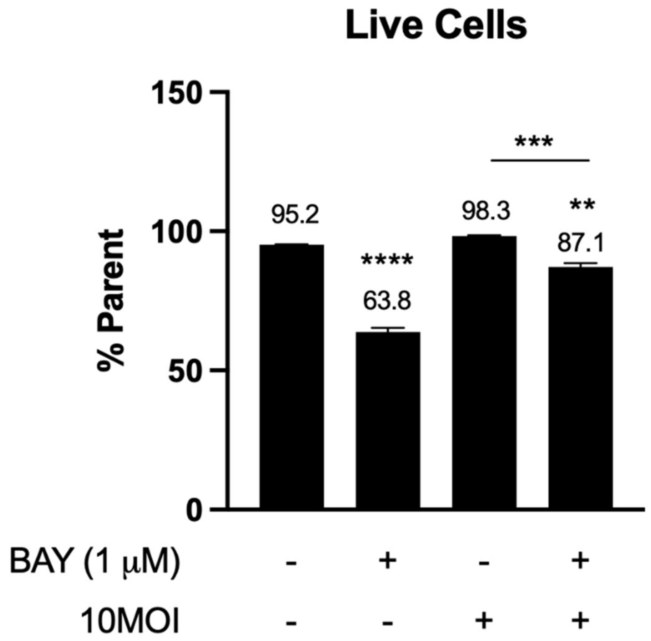

Figure S1. Cellular viability in the presence of BAY. Bone marrow-derived MΦ were treated with 1.0 μM BAY for 30 min prior to infection with *O. tsutsugamushi* (10 MOI). Viability was assessed at 6 hpi via live-dead staining. One-way ANOVA with Dunnett's multiple comparison test was performed across all treatment groups, using mock controls as the reference. **, $p < 0.01$; ***, $p < 0.0001$; ****, $p < 0.0001$.

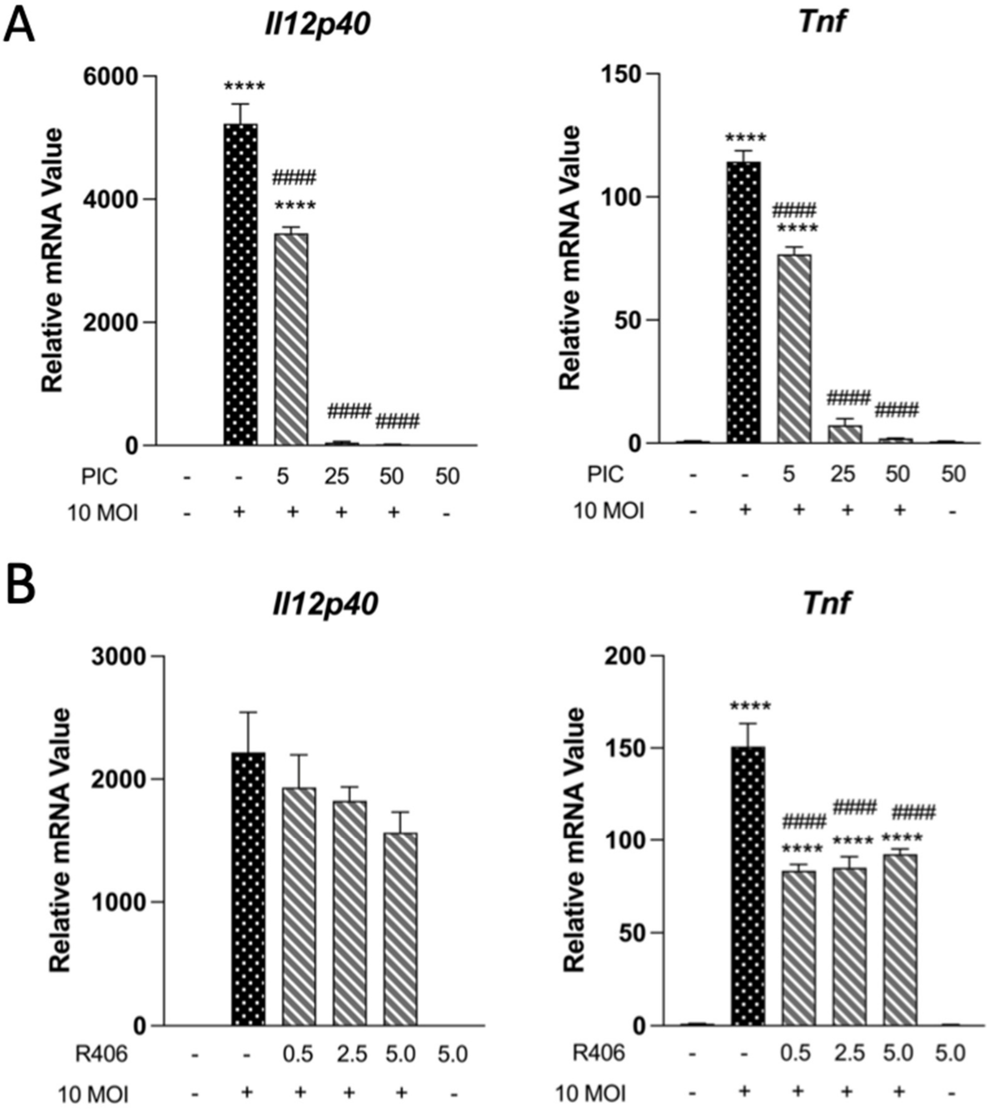

Figure S2. PIC and R406 exhibit different capacities to abrogate type 1 responses during infection. Bone marrow-derived MΦ were treated with Syk-inhibitors for 30 min prior to infection with *O. tsutsugamushi* (10 MOI). (A) Effect of PIC (5, 25, and 50 µM, respectively) on target genes at 6 hr post-infection. (B) Effect of R406 (0.5, 2.5, 5.0 µM) on target genes at 6 hr post-infection. qRT-PCR analyses of target genes relative to GAPDH are shown as mean ± SEM. One-way ANOVA with Dunnett's multiple comparison test was performed across all treatment

groups. For groups in comparison to mock controls: ****, $p < 0.0001$. For infected-and-treated groups in comparison to infected-but-untreated samples: ####, $p < 0.0001$.

Table S1. Primers for qRT-PCR analysis

Gene Target	Forward 5'-3'	Reverse 5'-3'
Clec4e (Mincle)	AGTGCTCTCCTGGACGATAG	CCTGATGCCTCACTGTAGCAG
Clec5a (MDL-1)	TCGGGGCTTATCGTAGTAGTG	TGTAGGCATGGTACTTTCGTCAT
Clec6a (Dectin1)	AAGCGGAGCAGAATTTCATCA	CCATTTGCCATTACCTTGTGGA
Clec12a	AGAAGTCTGACAAATGTGGGGG	CCAATGAACAGCAGAAGGCATA
Il12p40	GGAAGCACGGCAGCAGAATAA	CTTGAGGGAGAAGTAGGAATG
Il27p28	CTGTTGCTGCTACCCTTGCTT	CACTCCTGGCAATCGAGATTC
MerTK	CAGGGCCTTTACCAGGGAGA	TGTGTGCTGGATGTGATCTTC
Mx1	GACCATAGGGGTCTTGACCAA	AGACTTGCTCTTTCTGAAAAGCC
Oas1b	GGGCCTCTAAAGGGGTCAAG	TCAAACTTCACTCCACAACGTC
Oas2	TTGAAGAGGAATACATGCGGAAG	GGGTCTGCATTACTGGCACTT
Oas3	TCTGGGGTCGCTAAACATCAC	GATGACGAGTTCGACATCGGT
Tnf	CCCTCACACTCAGATCATCTTCT	GCTACGACGTGGGCTACAG
GAPDH	TGGAAAGCTGTGGCGTGAT	TGCTTCACCACCTTCTTGAT
